# Clinical Utility of the Combined Use of CA19-9 and DUPAN-2 in Pancreatic Adenocarcinoma

**DOI:** 10.1245/s10434-024-15221-z

**Published:** 2024-04-23

**Authors:** Tatsuaki Sumiyoshi, Kenichiro Uemura, Ryuta Shintakuya, Kenjiro Okada, Kenta Baba, Takumi Harada, Masahiro Serikawa, Yasutaka Ishii, Shinya Nakamura, Koji Arihiro, Yoshiaki Murakami, Shinya Takahashi

**Affiliations:** 1 Department of Surgery, Graduate School of Biomedical and Health Science, Hiroshima University, Hiroshima, Japan; 2 Department of Gastroenterology and Metabolism, Graduate School of Biomedical and Health Science, Hiroshima University, Hiroshima, Japan; 3 Department of Anatomical Pathology, Hiroshima University, Hiroshima, Japan; 4 Digestive Disease Center, Hiroshima Memorial Hospital, Hiroshima, Japan

## Abstract

**Purpose:**

Pancreatic ductal adenocarcinoma (PDAC) patients with normal carbohydrate antigen (CA) 19-9 levels can have early-stage cancer or advanced cancer without elevation of CA19-9 level; estimating their malignant potential is difficult. This study investigated the clinical utility of the combined use of preoperative CA 19-9 and Duke pancreatic monoclonal antigen type 2 (DUPAN-2) levels in patients with PDAC.

**Methods:**

Patients who underwent curative-intent surgery for PDAC between November 2005 and December 2021 were investigated. Eligible patients were classified into four groups based on these two markers. Among patients with normal CA19-9 levels, those with normal and high DUPAN-2 levels were classified into normal/normal (N/N) and normal/high (N/H) groups, respectively. Among patients with high CA19-9 levels, those with normal and high DUPAN-2 levels were classified into high/normal (H/N) and high/high (H/H) groups, respectively. Survival rates were compared between the groups.

**Results:**

Among 521 patients, the N/N, N/H, H/N, and H/H groups accounted for 25.0%, 10.6%, 35.1%, and 29.4% of patients, respectively. The proportions of resectable PDAC in the N/N and H/N groups (71.5% and 66.7%) were significantly higher than those in the N/H and H/H groups (49.1% and 54.9%) (*P* < 0.01). The 5-year survival rates in the N/N, N/H, H/N, and H/H groups were 66.0%, 31.1%, 34.9%, and 29.7%, respectively; the rate in the N/N group was significantly better than those in the other three groups (*P* < 0.0001, *P* < 0.0001, and *P* < 0.0001, respectively).

**Conclusions:**

Only patients with normal CA19-9 and DUPNA-2 values should be diagnosed with early-stage PDAC.

The most specific and sensitive biomarker for the prognosis of pancreatic ductal adenocarcinoma (PDAC) is the carbohydrate antigen (CA) 19-9.^1–9^ However, patients with PDAC with a Lewis antigen-negative phenotype secrete very little or no CA19-9.^10–13^ Therefore, in patients with PDAC with normal CA19-9 values, early-stage PDAC or advanced PDAC without elevation of CA19-9 levels can exist, making it difficult to estimate the malignant potential. To solve this problem, we assumed that the combined use of CA19-9 and Duke pancreatic monoclonal antigen type 2 (DUPAN-2) levels may be useful in estimating the malignant potential in all patients with PDAC, because DUPAN-2 levels do not depend on the Lewis antigen phenotype.^9,14,15^ Only a few previous studies have investigated the combined use of CA19-9 and DUPAN-2, because a few institutions measured DUPAN-2 levels routinely.^9,14^ However, our institution commenced routine measurements of both CA19-9 and DUPAN-2 in 2005, and this study includes the largest number of cases on this topic. The most important characteristic of this study is the cutoff values for CA19-9 and DUPAN-2. Several previous reports have demonstrated original institutional cutoff values of CA19-9 and DUPAN-2 to predict the prognosis of PDAC.^9,16,17^ These cutoff values were clinically important in each institution; however, it is unclear which cutoff value is the most appropriate. Whether these values are equally useful in all institutions is also a concern. Therefore, the cutoff values of CA19-9 and DUPAN-2 to distinguish between non-advanced and advanced PDACs were set to the upper normal limits (37 U/mL and 150 U/mL, respectively) in this study. Patients who underwent curative-intent surgery for PDAC were classified into four groups based on their preoperative CA19-9 and DUPAN-2 values, and the clinical utility of the combination of these two markers was investigated.

## Methods

### Study Design

Patients’ clinical data were collected through a retrospective review of prospectively maintained institutional databases. This study was approved by our institutional review board, and the requirement for informed consent was waived. All the procedures were performed in accordance with the principles of the 1964 Declaration of Helsinki and its later amendments or comparable standards.

### Patient Selection

Patients who underwent curative-intent surgery for PDAC at the Department of Surgery, Hiroshima University Hospital between November 2005 and December 2021 were investigated.

### Measurement of Tumor Markers

CA19-9 and DUPNA-2 levels were measured simultaneously in patients diagnosed with PDAC. Patients with jaundice underwent biliary drainage before any anticancer treatment, and tumor markers were measured after their serum total bilirubin level had reduced to <3.0 mg/dL.

### Classification of Eligible Patients

Normal serum CA19-9 level was defined as ≤37 U/mL and that of DUPAN-2 was ≤150 U/mL, as estimated based on the standard deviation in a normal population. The eligible patients were classified into four groups according to their upper normal limits. Among the patients with normal CA19-9 levels, those with normal and high DUPAN-2 levels were classified into normal/normal (N/N) and normal/high (N/H) groups, respectively. Among the patients with high CA19-9 levels, those with normal and high DUPAN-2 levels were classified into high/normal (H/N) and high/high (H/H) groups, respectively.

### Preoperative Treatment

Patients with borderline resectable PDAC received gemcitabine-based neoadjuvant chemotherapy. Patients with initially unresectable PDAC generally received gemcitabine plus nab-paclitaxel or FOLFIRINOX.^18–21^ No patient received preoperative radiation therapy.

### Surgical Procedures

The standard surgery for pancreatic head cancer was pylorus-preserving pancreaticoduodenectomy with lymphadenectomy.^22^ The standard surgical procedure for pancreatic body and tail cancer was distal pancreatectomy with lymphadenectomy.

### Pathological Diagnosis

A pathological diagnosis of PDAC was confirmed in all cases. Two experienced pathologists specializing in biliopancreatic malignancies confirmed the diagnosis using surgically resected specimens. Tumor stage and lymph node metastasis were classified based on the 8th edition of the Union for International Cancer Control (UICC) Tumor Node Metastasis Classification.

### Postoperative Follow-up

Patients routinely underwent computed tomography scans every three to four months after curative-intent surgery. We regarded patients as having a recurrence when the recurrent tumor was radiographically evident.

### Treatment Strategy After Recurrence

Systemic chemotherapy was the standard treatment when patients were diagnosed with recurrence during the postoperative follow-up. From December 2013, gemcitabine plus nab-paclitaxel was usually administered as first-line chemotherapy, considering the higher rates of grade 3–4 neutropenia and febrile neutropenia after administration of FOLFIRINOX in Japanese patients.^23^

### Outcome Measures

The following clinical parameters were investigated and compared between the groups: 1) proportion of patients; 2) clinicopathological features and overall survival rates; and 3) preoperative CA19-9 levels and correlations between CA19-9 and DUPAN-2 levels. Preoperative CA19-9 values were compared separately between patients with normal CA19-9 and high CA19-9 levels. The correlation between CA19-9 and DUPAN-2 levels was investigated separately in the patients with normal and high CA19-9 levels.

### Statistical Analysis

Median values were calculated, and nonparametric statistical testing procedures were used. Categorical variables were compared using the chi-square or Fisher’s exact test, as appropriate. Continuous variables were compared using the Mann–Whitney *U* test. For clinicopathological features, multiple comparisons were performed among the four groups. Additionally, we investigated the combinations of groups that were significantly different when statistical significance was detected among the four groups. Survival time was measured from the date of surgery to the date of death or last follow-up. Survival curves were established using the Kaplan–Meier method, and the significance of differences was evaluated using the log-rank test. Correlations between CA19-9 and DUPAN-2 values were analyzed using Spearman’s rank correlation test. All statistical analyses were performed using the JMP statistical software version 13 (SAS Institute, Cary, NC) under the supervision of a statistician. *P* < 0.05 was considered to indicate statistical significance.

## Results

### Eligible Patients

**Fig. 1:**
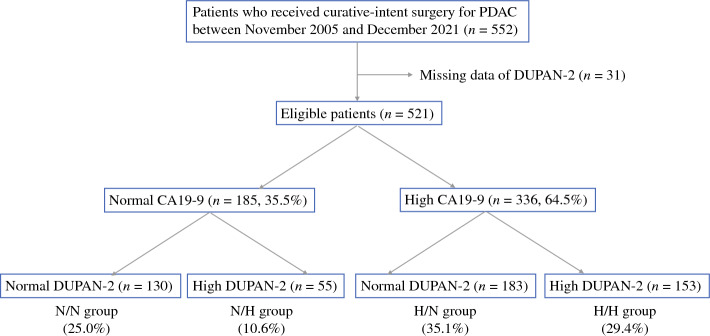
Patient flow chart. *CA19-9* carbohydrate antigen 19-9; *DUPAN-2* Duke pancreatic monoclonal antigen type 2; *N/N* normal CA19-9 and normal DUPAN-2; *N/H* normal CA19-9 and high DUPAN-2; *H/N* high CA19-9 and normal DUPAN-2; *H/H* high CA19-9 and high DUPAN-2

A total of 552 patients with PDAC underwent curative-intent surgery between November 2005 and December 2021. Of these, 31 patients who did not undergo DUPAN-2 measurements were excluded (Fig. 1). Among the remaining 521 patients, 185 (35.5%) patients had normal CA19-9 levels, and 336 (64.5%) patients had high CA19-9 levels. Among the patients with normal CA19-9 levels, 130 (25.0%) had normal DUPAN-2 levels (N/N group), and 55 (10.6%) had high DUPAN-2 levels (N/H group). Among the patients with high CA19-9 levels, 183 (35.1%) had normal DUPAN-2 levels (H/N group), and 153 (29.4%) had high DUPAN-2 levels (H/H group).

### Comparison of Clinicopathological Features

The clinicopathological features of patients in the four groups are shown in Table 1. Regarding preoperative factors, body mass index, sex, and tumor location did not significantly differ among the four groups. Patients in the H/H group were significantly younger than those in the H/N group. The proportions of patients with resectable PDAC in the N/N and H/N groups (71.5% and 66.7%, respectively) were significantly higher than those in the N/H and H/H groups (49.1% and 54.9%) (*P* < 0.01). Regarding surgery-related factors, surgery type, arterial resection rate, severe complication rate with Clavien–Dindo grade ≥3, and adjuvant chemotherapy administration rate did not significantly differ among the groups. The portal vein resection ratio, blood loss, and operation time in the N/N group were significantly smaller and shorter than those in the H/H group. All pathological factors were significantly advanced in the H/H group compared with the N/N group. The microvascular invasion rate and UICC N stage were significantly higher in the N/H group than in the N/N group. Lymphatic permeation rate, microvascular invasion rate, perineural invasion rate, and UICC N stage and R1 rate were significantly higher in the H/N group than in the N/N group.

**Table 1:** Patient characteristics in the four groups

Characteristics No. (%) or median (IQR)	N/N group (*n* = 130)	N/H group (*n* = 55)	H/N group (*n* = 183)	H/H group (*n* = 153)	*P*
Preoperative factors					
Age	71 (61–77)	69 (63–77)	72 (66–78)^a^	69 (62–75)^a^	0.035
BMI (kg/m^2^)	21.7 (19.6-23.9)	20.7 (18.9-23.2)	21.0 (19.1-23.3)	21.9 (19.3-23.8)	0.146
Sex (male)	75 (57.7)	26 (47.3)	103 (56.3)	89 (58.1)	0.544
Tumor location (Ph/Pb or Pt)	74 (56.9)/56 (43.1)	34 (61.8)/21 (38.2)	122 (66.7)/61 (33.3)	105 (68.6)	0.177
Resectability (R/BR or URLA)	93 (71.5)/37 (28.5)^b^	27 (49.1)/28 (50.9)^b,c^	122 (66.7)/61 (33.3)^c^	84 (54.9)/69 (45.1)^b,c^	<0.01
Surgery-related factors					
Surgery (PD/DP and TP)	76 (58.5)/54 (41.5)	34 (61.8)/21 (38.2)	123 (67.2)/60 (32.8)	103 (67.3)/50 (32.7)	0.339
Arterial resection	10 (7.7)	11 (20.0)	25 (13.7)	26 (17.0)	0.067
Portal vein resection	29 (22.3)^d^	21 (38.4)^d^	56 (30.6)	56 (36.6)^d^	0.043
Operation time (min)	295 (218-349)^e^	310 (240-415)	316 (241-379)	344 (270-425)^e^	<0.001
Blood loss (ml)	523 (296-852)^f^	610 (277-1200)	576 (323-1007)	773 (393-1525)^f^	<0.001
Complication (grade ≥3)	15 (11.6)	10 (18.2)	31 (16.9)	28 (18.3)	0.420
Adjuvant chemotherapy	98 (75.4)	45 (81.8)	141 (77.0)	113 (73.9)	0.673
Pathological factors					
Tumor differentiation (por)	7 (5.4)^g^	3 (5.5)	20 (10.9)	23 (15.0)^g^	<0.01
Lymphatic permeation	49 (37.7)^h^	23 (41.8)^i^	105 (57.4)^h,i^	92 (60.1)^h,i^	<0.001
Microvascular invasion	47 (36.2)^j^	27 (49.1)^j^	101 (55.2)^j^	89 (58.2)^j^	<0.01
Perineural invasion	92 (70.8)^k^	45 (81.8)	158 (86.3)^k^	137 (89.5)^k^	<0.001
UICC T stage (T3, T4)	23 (17.7)^l^	10 (18.2)	44 (24.0)	48 (31.4)^l^	0.038
UICC N stage (N0/N1/N2)	69 (53.1)/42 (32.3)/ 19 (14.6)^m^	16 (29.1)/29 (52.7)/ 10 (18.2)^m^	65 (35.5)/72 (39.3)/ 46 (25.1)^m^	36 (23.5)/70 (45.8)/ 47 (30.7)^m^	<0.001
Residual tumor R0/R1	113 (86.9)/17 (13.1)^n,o^	45 (81.8)/10 (18.2)^n^	143 (78.1)/40 (21.9)^n,o^	101 (66.0)/52 (34.0)^n^	<0.001

*IQR* interquartile range; *N/N* normal carbohydrate antigen 19-9 (CA19-9) and normal *Duke Pancreatic monoclonal antigen type 2* (DUPAN-2); *N/H* normal CA19-9 and high DUPAN-2; *H/N* high CA19-9 and normal DUPAN-2; *H/H* high CA19-9 and high DUPAN-2; *BMI* body mass index; *Ph* pancreas head; *Pb* pancreas body; *Pt* pancreas tail; *R* resectable; *BR* borderline resectable; *URLA* initially unresectable locally advanced; *PD* pancreaticoduodenectomy; *DP* distal pancreatectomy; *TP* total pancreatectomy; *Grade ≥3* Clavien-Dindo grade ≥3; *por* poorly differentiated adenocarcinoma

^a-o^Statistical significances between the specific groups (*P* < 0.05): ^a^H/N *vs.* H/H; ^b^N/N vs. N/H, N/N vs. H/H; ^c^H/N vs. N/H, H/N vs. H/H; ^d^N/N vs. N/H, N/N vs. HH; ^e^N/N vs. H/H; ^f^N/N vs. H/H; ^g^N/N vs. H/H; ^h^N/N vs. H/N, N/N vs. H/H; ^i^N/H vs. H/N, N/H vs. H/H; ^j^N/N vs. N/H, N/N vs. H/N, N/N vs. H/H; ^k^N/N vs. H/N, N/N vs. H/H; ^l^N/N vs. H/H; ^m^N/N vs. N/H, NN vs. H/N, NN vs. HH; ^n^H/H vs. N/N, H/H vs. N/H, H/H vs. H/N; ^o^N/N vs. H/N

### Overall Survival Rate

**Fig. 2:**
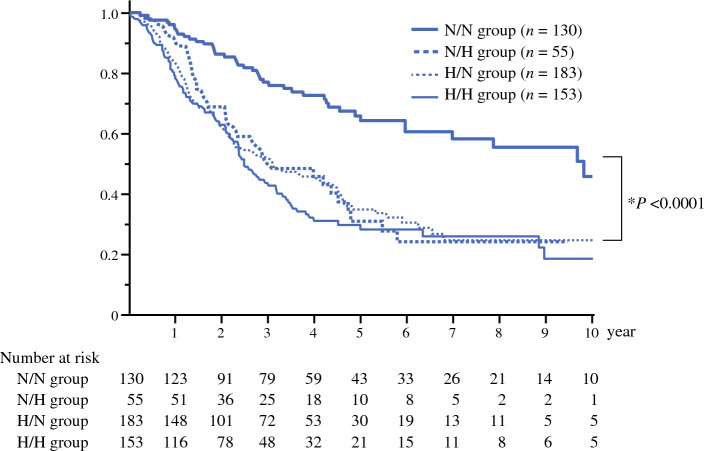
Survival curves of patient groups. *N/N* normal CA19-9 and normal DUPAN-2; *N/H* normal CA19-9 and high DUPAN-2; *H/N* high CA19-9 and normal DUPAN-2; *H/H* high CA19-9 and high DUPAN-2. *N/N vs*.* N/H, N/N vs*.* H/N, and N/N vs*.* H/H

The median survival times in the N/N, N/H, H/N, and H/H groups were 119.7, 36.9, 38.2, and 30.6 months, respectively (Fig. 2). The 5-year survival rates were 66.0%, 31.1%, 34.9%, and 29.7%, respectively. The survival curves were similar in the N/H, H/N, and H/H groups, and no significant differences in survival rates were observed among the three groups (N/H vs*.* H/N, *P* = 0.871; N/H vs. H/H, *P* = 0.364; H/N vs*.* H/H, *P* = 0.335). However, the survival rate in the N/N group was significantly higher than that in the other three groups (*P* < 0.0001, *P* < 0.0001, and *P* < 0.0001).

### Comparisons of CA19-9 Values

**Fig. 3:**
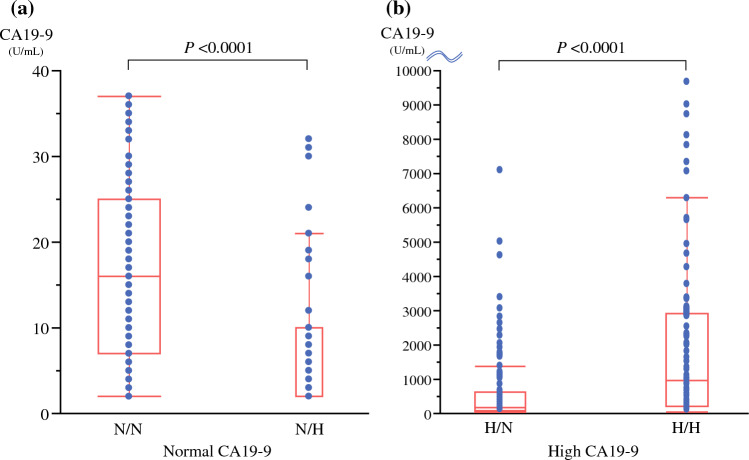
Comparison of CA 19-9 values. **a** Comparison of CA 19-9 values in patients with normal CA19-9 levels. CA19-9 levels were significantly lower in the N/H group than in the N/N group (*P* <0.0001). **b** Comparison of CA 19-9 values in patients with high CA19-9 levels. CA19-9 levels were significantly higher in the H/H group than in the H/N group (*P* <0.0001). *N/H* normal CA19-9 and high DUPAN-2; *N/N* normal CA19-9 and normal DUPAN-2; *H/H* high CA19-9 and high CA19-9; *H/N* high CA19-9; normal DUPAN-2; *CA19-9* carbohydrate antigen 19-9; *DUPAN-2* Duke pancreatic monoclonal antigen type 2

In patients with normal CA19-9 levels, the CA19-9 levels were significantly lower in the N/H group than in the N/N group (median 16.0 U/mL [N/N] vs*.* 2.0 U/mL [N/H], *P* < 0.0001) (Fig. 3a). In patients with high CA19-9 levels, the CA19-9 levels were significantly higher in the H/H group than in the H/N group (median 187 U/mL [H/N] vs*.* 942 U/mL [H/H], *P* < 0.0001) (Fig. 3b).

### Correlation Between CA19-9 and DUPAN-2 Values

**Fig. 4:**
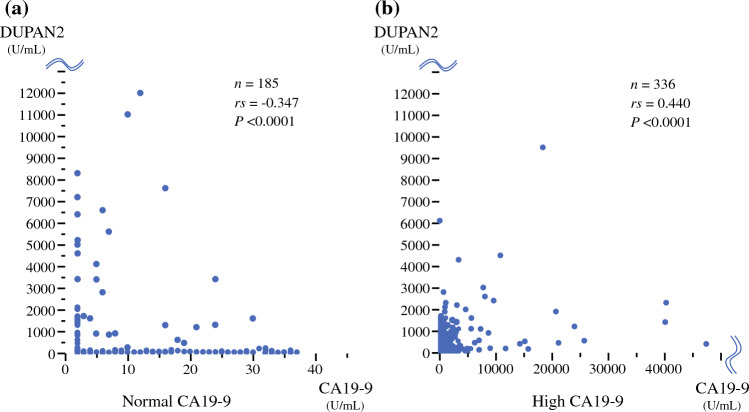
Correlation between CA19-9 and DUPAN-2. **a** In patients with normal CA19-9 values, a very weak negative correlation was observed between CA19-9 and DUPAN-2 values (correlation coefficient, −0.347; *P* < 0.0001). **b** In patients with high CA19-9 values, a very weak positive correlation was observed between CA19-9 and DUPAN-2 levels (correlation coefficient, 0.440; *P* < 0.0001)

The correlation between CA19-9 and DUPAN-2 levels in patients with normal and high CA19-9 levels is presented in Fig. 4. In patients with normal CA19-9 levels, a very weak negative correlation was observed between CA19-9 and DUPAN-2 levels (correlation coefficient: −0.347; *P* < 0.0001) (Fig. 4a). In patients with high CA19-9 levels, a very weak positive correlation was observed between CA19-9 and DUPAN-2 levels (correlation coefficient: 0.440; *P* < 0.0001) (Fig. 4b).

## Discussion

CA19-9 is a monoclonal antibody to the Lewis (a) antigen associated with tumor progression; it is the most commonly used biomarker for PDAC, with a sensitivity of approximately 80%.^1–3,5–7,24,25^ NCC guidelines recommend CA19-9 in screening, diagnosis, staging, determining resectability, and monitoring therapeutic response.^26^ However, CA19-9 is not always useful in patients with PDAC. Approximately 5–10% of patients have a Lewis antigen-negative phenotype and secrete little to no CA19-9.^10–13^ Regarding the CA19-9 value in Lewis-negative patients with PDAC, it has recently been reported that CA19-9 levels are not always undetectable. Luo et al. reported that 72.6% of Lewis-negative patients had normal CA19-9 levels (<37 U/mL). However, only 41.9% of the patients had undetectable CA19-9 levels (<2 U/mL). Therefore, it is difficult to detect Lewis-negative patients among patients with normal CA19-9 levels using only CA19-9 levels; estimating the malignant potential in patients with normal CA19-9 levels also is challenging.

To address this issue, we investigated the clinical utility of the combined use of CA 19-9 and DUPAN-2. DUPAN-2 is a monoclonal antibody to Lewis (c); it is converted to Lewis (a) by *α*-1-3/4 fucosyltransferase, the Lewis enzyme.^9^ This marker has an advantage, which can be used irrespective of the Lewis antigen phenotype.^9,14,15^ In Lewis-negative patients, CA19-9 levels are typically not elevated; however, DUPAN-2 accumulates in the serum due to the absence of *α*-1-3/4 fucosyltransferase.^13,27^ Conversely, DUPAN-2 has disadvantages of lower sensitivity and prognostic value compared to CA19-9,^8^ leading to the concept of combining both markers in this study.

The investigation of CA19-9 and DUPAN-2 revealed the proportion of patients in each group, the CA19-9 levels in each group, the correlation between CA19-9 and DUPAN-2, and the clinical characteristics and survival period of patients in each group.

First, more than one third of patients had normal preoperative CA19-9 levels, and estimating the malignant potential using only CA19-9 was difficult in those patients. Furthermore, approximately half of the all patients had discrepancies in the normality and abnormality of CA19-9 and DUPAN-2 values (N/H group: 10.6%, H/N group: 35.1%), indicating that the two markers have different clinical features.

Second, the trends in CA19-9 values significantly differed between the patients with normal and high CA19-9 levels. Therefore, the correlation between CA19-9 and DUPAN-2 should be considered after classifying patients into normal and high CA19-9 groups. In patients with normal CA19-9 levels, the values were significantly lower in the N/H group than in the N/N group, indicating a negative correlation between CA19-9 and DUPAN-2 levels. In contrast, in patients with high CA19-9 levels, the values were significantly higher in the H/H group than in the H/N group, indicating a positive correlation between CA19-9 and DUPAN-2 levels. Several previous studies reported no correlation between the two markers; however, these studies investigated the correlation in all patients with PDAC without classifying patients based on the normality of the CA19-9 value.^8,14,28^ Only one study from the Shizuoka Cancer Center group reported a correlation after categorizing patients by CA19-9 values. Similar to our study, in patients with normal CA19-9 values, only a very weak negative correlation was observed between CA19-9 and DUPAN-2 levels; in patients with high CA19-9 values, a positive correlation was observed.^9^ This negative correlation in patients with normal CA19-9 values may be caused by the Lewis-negative phenotype. Most Lewis-negative patients have normal CA19-9 values, and DUPAN-2 values can be higher because Lewis (c) is either not converted to Lewis (a) or is converted in only a small amount.^25,29^ The more aggressively the tumor produces Lewis (c) in Lewis-negative patients, the greater the discrepancy between CA19-9 and DUPAN-2 values.

Third, the most important finding of this study was the remarkable difference in the survival period between the groups. The five-year survival rates in the N/H, H/N, and H/H groups were approximately 30%, with similar survival curves. In contrast, the 5-year survival rate in the N/N group was 66.0%, which was significantly higher than that in the other three groups (*P* < 0.0001, *P* < 0.0001, and *P* < 0.0001, respectively). Two previous studies have investigated the combined use of the two markers and the survival period. The Oita University group investigated 87 patients who underwent surgical resection for PDAC and assigned the patients to four groups according to normal or high levels of the two markers.^14^ Unlike our findings, only patients in the H/H group had significantly poorer survival than those in the other three groups. However, the number of patients in that study was small. Another study from the Shizuoka Cancer Center investigated 224 patients and assigned them to N/N, NH, and high CA19-9 groups. The cutoff value of CA19-9 was set to the upper normal limit (37 U/mL), whereas that of DUPAN was set to the original value of 250 U/mL. Although the cutoff value of DUPAN-2 and classification of patients differed from those in our study, only patients in the N/N group showed significantly better survival than those in the other groups, similar to our study’s findings.

Our study included the largest sample size, and by including the four groups, especially the N/H and H/N groups, the following hypotheses were proposed. When the tumor becomes aggressive, Lewis (a) is actively produced to promote invasion and metastasis,^24^ and the CA19-9 value is elevated in Lewis-positive patients. However, in Lewis-negative patients, Lewis (c) and not Lewis (a) accumulates, leading to an elevation of DUPAN-2 levels. Therefore, the elevation of either CA19-9 or DUPAN-2 is assumed to indicate an elevation in malignant potential. Because Lewis (c) is the precursor of Lewis (a), DUPAN-2 can be considered an approximation of CA19-9. However, CA19-9 and DUPAN-2 should be considered different biomarkers that compensate for each other.

Furthermore, investigating the clinicopathological features and survival curves of the four groups revealed a novel important feature. The resectability statuses were similar between the N/N and H/N groups (resectable PDAC: 71.5% vs*.* 66.7%, respectively); however, the microvascular invasion rate and UICC N stage and R1 rate were significantly higher in the H/N group, and the survival period was significantly longer in the N/N group. The resectability status is related only to the tumor size and location and may occasionally not indicate the actual malignant potential; therefore, a great discrepancy in survival outcome was found between the N/N and H/N groups, despite having similar resectability statuses. The preoperative measurement of both CA19-9 and DUPAN-2 can offer a more accurate evaluation of the malignant potential; it can extract low and highly malignant PDACs among the normal CA19-9 groups. The findings of this study can fundamentally change treatment strategies for PDAC. Patients in the three groups other than the N/N group might be appropriate to be treated as having biological borderline resectable PDAC.

This study has some limitations. First, this study was based on data from a single-center database, and unexpected bias cannot be completely excluded, although it included the largest number of cases. Second, most Lewis-negative patients were surmised to belong to the NH group; however, the actual CA19-9 and DUPAN levels in these patients were not investigated. To address this problem, we plan to measure the Lewis antigen phenotype in patients with PDAC in further studies.

In conclusion, only patients with normal CA19-9 and DUPNA-2 values should be considered to have early-stage PDAC.

## Data Availability

The data are not publicly available due to their containing information that could compromise the privacy of research participants.
